# PANI and Graphene/PANI Nanocomposite Films — Comparative Toluene Gas Sensing Behavior

**DOI:** 10.3390/s131216611

**Published:** 2013-12-03

**Authors:** Mitesh Parmar, Chandran Balamurugan, Dong-Weon Lee

**Affiliations:** 1 MEMS and Nanotechnology Laboratory, School of Mechanical Systems Engineering, Chonnam National University, Gwangju 500757, Korea; E-Mails: miteshparmar17@gmail.com (M.P.); cbalamurugan2008@gmail.com (C.B.); 2 Department of Instrumentation and Applied Physics, Indian Institute of Science, Bangalore 560012, India

**Keywords:** graphene, PANI, nanocomposite polymer, toluene, sensing

## Abstract

The present work discusses and compares the toluene sensing behavior of polyaniline (PANI) and graphene/polyaniline nanocomposite (C-PANI) films. The graphene–PANI ratio in the nanocomposite polymer film is optimized at 1:2. For this, N-methyl-2-pyrrolidone (NMP) solvent is used to prepare PANI-NMP solution as well as graphene-PANI-NMP solution. The films are later annealed at 230 °C, characterized using scanning electron microscopy (SEM) as well Fourier transform infrared spectroscopy (FTIR) and tested for their sensing behavior towards toluene. The sensing behaviors of the films are analyzed at different temperatures (30, 50 and 100 °C) for 100 ppm toluene in air. The nanocomposite C-PANI films have exhibited better overall toluene sensing behavior in terms of sensor response, response and recovery time as well as repeatability. Although the sensor response of PANI (12.6 at 30 °C, 38.4 at 100 °C) is comparatively higher than that of C-PANI (8.4 at 30 °C, 35.5 at 100 °C), response and recovery time of PANI and C-PANI varies with operating temperature. C-PANI at 50 °C seems to have better toluene sensing behavior in terms of response time and recovery time.

## 1. Introduction

One of the main reasons for the vast advancement in the field of sensing technology is to provide safety and security to mankind. Air pollution influences human health and can cause a number of diseases. The major air pollutants include CO/CO_2_, NO*_x_*, SO_2_ and volatile organic compounds (VOCs). The main VOCs contributing to pollution are benzene, toluene, ethylbenzene and xylenes—commonly known as BTEX. Among BTEX, benzene is one of the most commonly used substances in many chemical and process industries for manufacturing rubber, lubricants, dye, detergents, drugs, pesticides, *etc*., [1,2]. However, benzene, being carcinogenic, is often replaced with alternate chemicals like toluene. Nevertheless, human exposure to higher concentrations of toluene can still be hazardous and life-threatening. According to the UK Health Protection Agency (HPA), the occupational standard for 8 h toluene exposure is 50 ppm (191 mg/m^3^) [3]. Therefore, there is an increasing need for efficient toluene sensors to monitor and control the emissions of toluene.

Based on the sensing mechanism, sensors can be categorized as resistive sensors, quartz crystal-based sensors, surface acoustic wave (SAW)-based sensors and also field-effect transistor (FET)-based (which shows device characteristics change) sensors [4]. Due to the inherent advantage of resistive-based sensors, such as high sensitivity and easy circuitry, they are the most widely researched toluene sensors. Table 1 shows the sensing behavior of some of the resistive-based toluene sensors reported in the recent times. As can be observed from the table, intrinsically conductive polymers (ICPs) are not as widely used as active sensing material for toluene detection compared to other strong oxidizing or reducing gases. Although the limit of detection (LOD) for metal oxide (MOX)-based sensors is generally better (up to parts per billion *i.e.*, ppb); their operating temperature is comparatively much higher than that of ICPs. For MOXs, toluene dehydrogenates at the sensing surface and this alters the work function of the sensing film by donating electrons and changing the Fermi level [5–7]. Depending on the type of semiconducting MOX used, the film resistance increases or decreases in the presence of analyte. The case with ICPs is similar. In the case of ICPs, the sensor output is based on the variation in conductivities due to the change in work functions [8]. However, these ICPs generally respond in similar way towards different analytes. This problem can be overcome by tuning these ICPs, which helps to prepare a variety of sensing films. Incorporation of other micro/nanoparticles helps to obtain conductive polymer nanocomposites (CPCs) and to enhance their selectivity. Some of the recent works on CPCs exhibit not only improvements in selectivity, but also in LOD, even for room temperature operation [9,10]—one of the main drawbacks of the MOX sensors.

Recently, a different sensing mechanism was proposed by Matsuguchi *et al.* [11] for toluene sensing using carbon black–N,N-dimethyl-1,3-propanediamine (MCD) co-polymer. According to this mechanism, a change in the resistance of the sensing material is observed due to breakdown of the conducting network as a result of sorption at insulating toluene into micro-voids. However, there is a constant negative shift in the base resistance value at every sensing cycle of 200 ppm toluene. This shift in the base resistance line can be due to non-reversible accumulation of analyte or chain relaxation. As the sensing mechanism is resistive-based, the equal change in the resistance value exhibits a shift in sensitivity due to the varying resistance baseline. Considering the advantages offered by ICP-based sensors such as, low cost and possibility of working at room temperatures besides their processing simplicity; ICPs can play a vital role in room temperature toluene sensing, unlike MOXs in Table 1. Nevertheless, ICP-based toluene sensors need further improvement before their commercialization owing to insufficient reproducibility, sensitivity to humidity, temporal drift of specific conductivity and their susceptibility to poisoning.

Polyaniline (PANI) is one of the most technologically promising ICPs. Its advantages include easy synthesis, environmental stability, low cost, controllable electrical conductivity, and interesting redox properties [19–26]. In order to overcome some of the above-mentioned limitations of ICPs, PANI is used heterogeneously along with different materials to form conductive polymer nanocomposites (CPCs with ICP matrix). As discussed by Stankovich *et al.* [27], the properties of any CPC largely depend on the aspect ratio and surface-to-volume ratio of the filler. Graphene (GR), being a 2D material, possesses excellent surface-to-volume ratio. In addition to this, it has some of the unique characteristics such as excellent carrier mobility (∼10,000 cm^2^·V^−1^·s^−1^), very high surface to volume ratio (theoretically 2630 m^2^·g^−1^), thermal conductivity (3000–5000 W·m^−1^·K^−1^), Young's modulus (0.5–1 TPa) and ultimate strength of 130 GPa, low Johnson as well as 1/f noise (switching) due to few crystal defects, *etc.* —is another wonderful material that has enthralled researchers worldwide [27–32].

According to the literature, the pristine GR is not suitable for gas sensing applications because of low adsorption energies of test gas molecules on the GR surface [22,30,33]. Hence, GR is functionalized with elements such as B, N, Al, Si, Cr, Mn, Pd, Pt, Ag, Au, or other metal common gas sensing materials such as ZnO, WO_3_ and TiO_2_ [27–32,34–36]. In addition to this, GR is also used with polymer ionic liquid (PIL) for sensing application [9]. The incorporation of GR in polymer *i.e.*, graphene polymer nanocomposite (Gr-PnC) is a way to get best of both materials—GR and polymer. A composite is a combination of multiple materials in which the property might be a weighted average of the components or a completely new one. The recent studies discuss the numerous applications along with structural, optical, thermal and electrical properties of Gr-PnC [27,34–36]. These composites contain GR with different polymer matrix. The polymer used in these matrixes can be either intrinsically conducting polymer (ICP) or non-conducting polymer (NCP). Depending on the kind of polymer matrix, the interaction between these composites and analyte vapor varies.

Owing technological promises of PANI, graphene/PANI nanocomposite (C-PANI) is attracting interest of scientific community [10,33,36–43]. Yet the studies on sensing property of C-PANI started recently [39–41]. Therefore, we are reporting the comparative sensing behavior of intrinsic PANI and GR/PANI nanocomposite film towards toluene gas. For this, the polymer nanocomposite films are grown using spin coating. In order to compare the sensing behavior of nanocomposite PANI films with homogeneous PANI films, the PANI based sensors are also fabricated following the similar technique. The films are characterized using scanning electron microscopy (SEM) as well as Fourier transform infrared spectroscopy (FTIR) and later are analyzed at different operating temperature for the sensing of 100 ppm toluene.

## 2. Experimental Section

### 2.1. Fabrication of PANI and C-PANI Based Sensor

The PANI (emeraldine salt; Sigma Aldrich, St. Louis, MO, USA) is first converted into the base form by treating it with ammonia (NH_4_OH) solution and later dissolved in N-methyl-2-pyrrolidone (NMP; Sigma Aldrich) by a combined magnetic stirring and sonication process. After dissolving the PANI in NMP, the solution is divided into two parts. To one of the parts, graphene is added to make graphene-PANI in 1:2 ratio. The nanocomposite PANI-NMP solution is further stirred magnetically and sonicated (at 200 watt for 6 h) to uniformly disperse the graphene flacks. The homogeneous PANI-NMP solution and nanocomposite PANI-NMP (*i.e.*, GR-PANI-NMP) solution are tagged as Sol1 and Sol2 respectively. The films of these solutions are spin coated layer-by-layer (LbL) on piranha-cleaned SiO_2_-coated Si substrates. In order to improve the adhesion of the polymer films towards the SiO_2_-coated Si substrate, hexamethyldisilazane (HMDS) is used as interfacial layer. The films are spin-coated LbL ten times in order to obtain a measurable resistance using a Sourcemeter 2400 (Keithley Instruments, Inc., Solon, OH, USA). The thickness of sensing film is observed to be ∼500 nm, measured using cross-section SEM imaging. The samples spin-coated using solutions Sol1 and Sol2 are named S1 and S2, respectively. After the coating of the polymer films, they are kept at 230 °C for 3 h to remove moisture and NMP solvent. The heating temperature is selected after a careful examination of the respective material properties of NMP and PANI.

**Figure.1.**
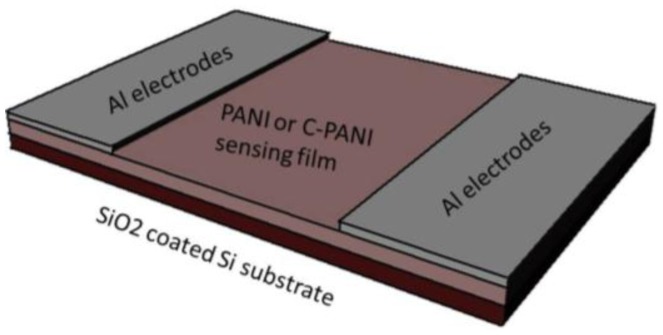
Schematic diagram of a toluene sensor.

In order to study the electrical characteristics and the sensing behavior, electrode deposition is the next important step. The electrode deposition, in a two-end configuration, is performed by direct current (DC) sputtering of aluminum (Al) over the S1-S2 samples. Al is sputtered for 60 min at 57–59 W power. The Al film thickness is measured using an optical surface profilometer (NanoSystem NV-1000 equipped with NanoView software, NanoSystem Co. Ltd., Daejeon, Korea) as well as mechanical testing (Veeco Dektak 150 with DektekV9 software, Veeco Instruments Inc., New York, NY, USA), and found to be ∼100 nm. After the electrode deposition, the wire connection is taken using conductive epoxy (Circuitworks^®^ conductive epoxy CW2400, ITW Chemtronics, Kennesaw, GA, USA). The schematic of the sensor is shown in Figure 1. These polymer films are characterized using SEM and FTIR. The FTIR characterization is performed in the wavenumber range from 450 to 4000 cm^−1^, using a Frontier FT-IR/NIR instrument (PerkinElmer, Waltham, MA, USA).

### 2.2. Sensor Testing Setup

**Figure 2.**
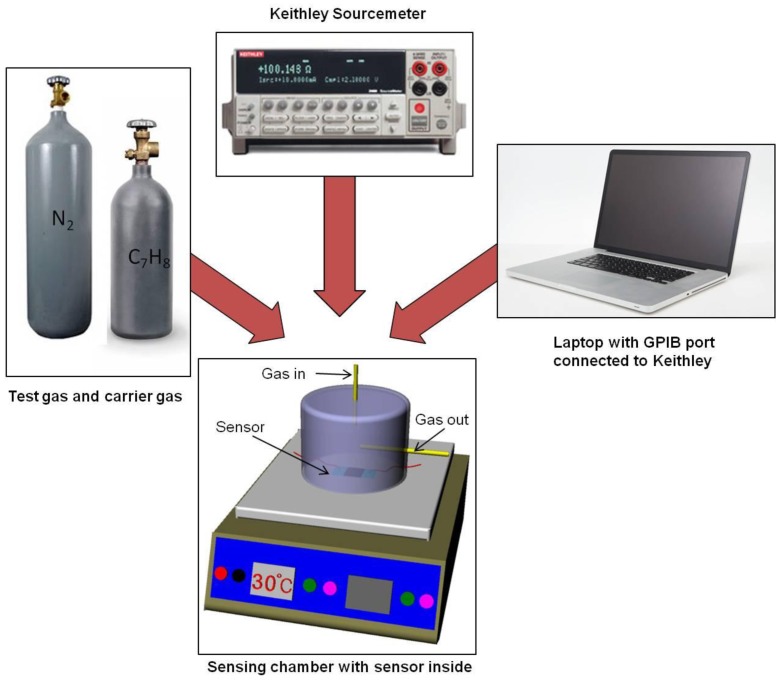
Schematic representation of the sensor testing setup.

The samples S1 and S2 are analyzed for their sensing behavior using an in-house developed cost-effective as well as reliable gas sensor testing setup. The sensor testing setup consists of a laboratory hot-plate, an acrylic glass chamber with inlet-outlet and pressure monitoring outlet along with electrical connections, mass flow controllers (Bronkhorst-Hightech model F201CV, Bronckhorst-Hightech, Gelderland, The Netherlands) and Keithley Sourcemeter 2400. The sourcemeter is interfaced with the computer using IEEE-488 General Purpose Interface Bus (GPIB) cable and real time data recording is performed with the help of a LabVIEW-based program (National Instruments, Austin, TX, USA). The gas-sealing is made of polydimethylsiloxane (PDMS) using a conventional molding technique [44]. Figure 2 shows the sensor testing setup.

The electrical analyses are performed by taking I-V characteristics and R-V characteristics for the voltage ranges of 0 to +10V/−10V along with the hysteresis loop, at the operating temperature of 30 °C. Later, the sensing analysis on these samples is performed with temperature modulation. The sensing analysis includes sensor response, response time and recovery time.

## 3. Results and Discussion

In the present study, two types of sensing films are under consideration—PANI (ICP) and C-PANI (CPC with ICP matrix) films. As discussed before, the interaction of analyte with ICPs and with CPCs + ICPs using ICP matrix will be different. In case of ICPs, the sensor output will be based on the variation in conductivities due to a change in work functions [8]. For the CPC (with a non-conducting polymer matrix), the variation in the film conductivities will be related to changes in the tunnelling conduction of the percolated network. This tunnelling conduction is a function of the film swelling due to adsorbance of analyte molecules at filler/filler junctions [8,42,43]. In case of a CPC with an ICP matrix, it can be a combination of both. Before discussing further the sensing mechanism or the sensing behaviour, the sensing film and the parameters for the formation of the GR-PANI matrix need to be considered.

### 3.1. Material Characterization

**Figure 3.**
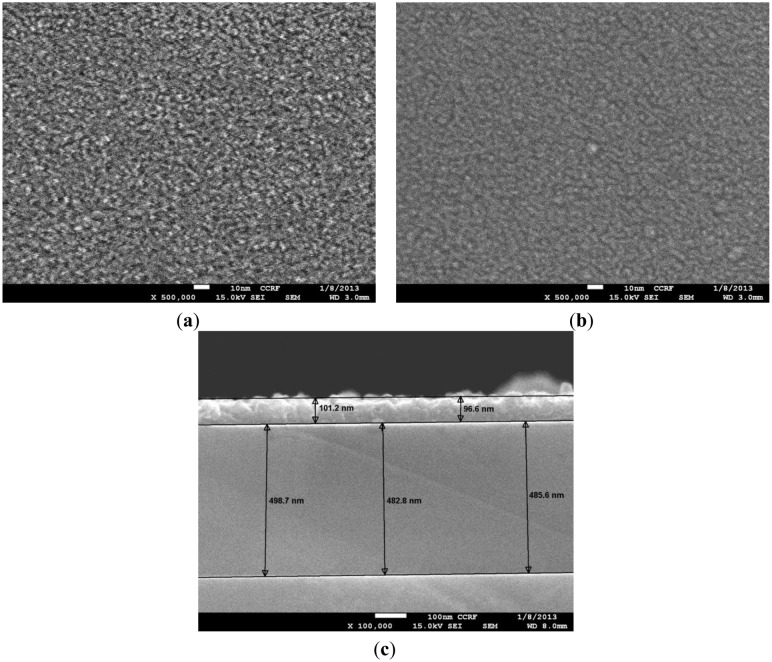
(**a**,**b**) SEM images showing the morphology of PANI and C-PANI; (**c**) Cross-sectional SEM image of polymer sample for thickness measurement.

The SEM images in Figure 3a–c show the surface morphology of PANI and nanocomposite C-PANI along with the cross-sectional SEM image of spin-coated polymer film annealed at 230 °C. Figure 3b does not exhibit any GR flakes for the C-PANI sample since the GR is dispersed in the PANI matrix and not over the surface. Nevertheless, the presence of GR in C-PANI can be shown by the FTIR peak shifts and changes in film conductivity or the baseline resistance. The variation between the expected thickness and measured thickness is due to the annealing. The thickness of PANI films are found to be ∼500 nm, whereas the thickness of Al film is ∼100 nm.

**Figure 4.**
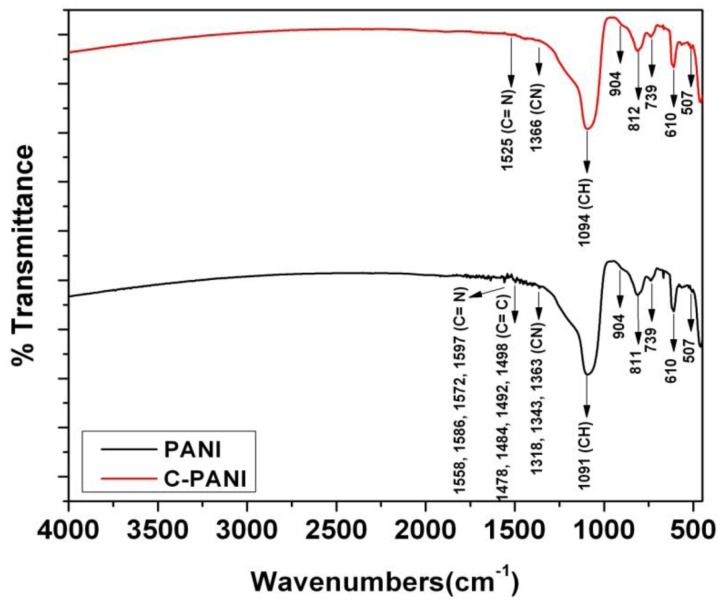
FTIR analysis of PANI and C-PANI material.

The polymers films—PANI and C-PANI are analyzed using FTIR, as shown in Figure 4. The major peaks for PANI in this spectrum belongs to CH vibrations, CN vibrations, C=C vibrations and C=N vibrations and coincides with the literature as well [45].

The initial peaks in PANI and C-PANI such as 507 and 610 are due to aromatic ring deformation. However, the shift in C-PANI for CH, CN, C=C and C=N vibrations is visible due to graphene—PANI interaction in the PANI matrix.

### 3.2. Sensing Behavior

As the sensing mechanism is based on the variation of conductivity, electrical characterization is quite necessary. The increase or decrease in the conductivities will depend on the negative vapor coefficient (NVC) or positive vapor coefficient (PVC) effect. In the PVC case, a sharp increase of film resistance is observed in the presence of analyte vapor. The opposite occurs in the case of the NVC effect. Here, nitrogen (N_2_, 99.99%) gas is used as carrier gas and 100 ppm toluene (C_6_H_5_–CH_3_) gas containing air as analyte. Prior to sensing studies, the sensing chamber is flushed with N_2_ gas. During N_2_ gas flushing, the resistance of both the sensing films increased. This can be due to desorption of the adsorbed gases and the stabilization of the base resistance. After that, the toluene-containing air (the analyte or test gas in our case) is admitted to the chamber. For the sample S1, upon the admission of toluene gas, the resistance of the sensing film increases, confirming the general polymer sensing mechanism and its PVC effect. However, the PVC effect displayed by the S1 sample is quite irregular and hence it raises doubts about the reliability of the data. Nevertheless, heat treatment of these samples at 100 °C in a CO_2_ environment for 6–8 h shows the NVC effect. No such change is observed for the C-PANI sample. The detailed sensing mechanism is discussed in the next section.

**Figure 5.**
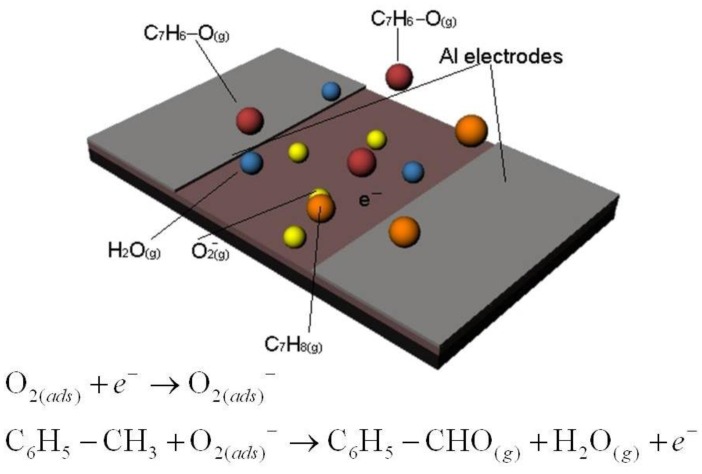
Toluene sensing mechanism.

After the stabilization of the base resistance during N_2_ flushing, 100 ppm toluene-containing air is admitted into the sensing chamber. Considering the sensing behavior discussion of ICP and CPC (with ICP matrix), it can be said that toluene is dehydrogenated and in the process releases free electrons in the PANI (ICP) sensing film. This in turn changes the work functions as well as the conductivity of the ICP. Figure 5 shows the sensing mechanism along with the probable chemical reactions. Along with this effect for CPCs + ICPs, the presence of active sites on the graphene in C-PANI and swelling junctions between graphene flakes facilitates further chemisorption of toluene vapor. As a result, C-PANI films show enhanced sensing behavior.

The sensor shows response towards toluene by exhibiting NVC effect. After admitting toluene-containing air for 10 min to facilitate the continuous dehydrogenation, the residual gases are flushed using N_2_ gas again. The selection of 10 min as waiting time is due to the inability of reaching a saturated resistance change (ΔR). The flow rate of N_2_ gas is maintained at 1 standard cubic centimeter per minute (SCCM).

**Figure 6.**
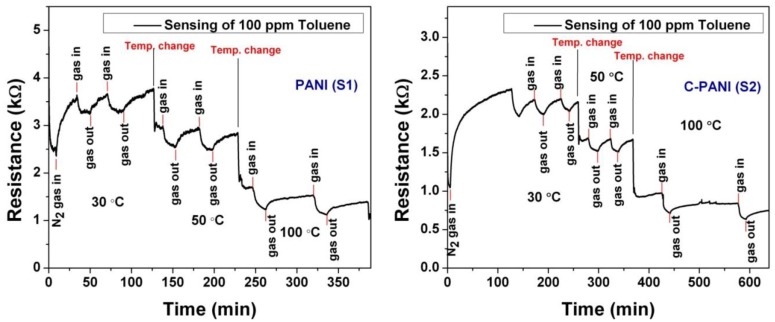
The toluene sensing behavior of PANI (S1) and C-PANI (S2) films at different operating temperatures (30, 50 and 100 °C).

The behaviors of both the samples (PANI and C-PANI) at different operating temperatures (30, 50 and 100 °C) are shown in Figure 6. However, the poor reversibility of sensor at 100 °C is under consideration. We came to the conclusion that the poor reversibility might be due to the chemisorption of toluene vapor at 100 °C and the inability for complete desorption of residual gases with N_2_ gas flow at 1 SCCM.

**Figure 7.**
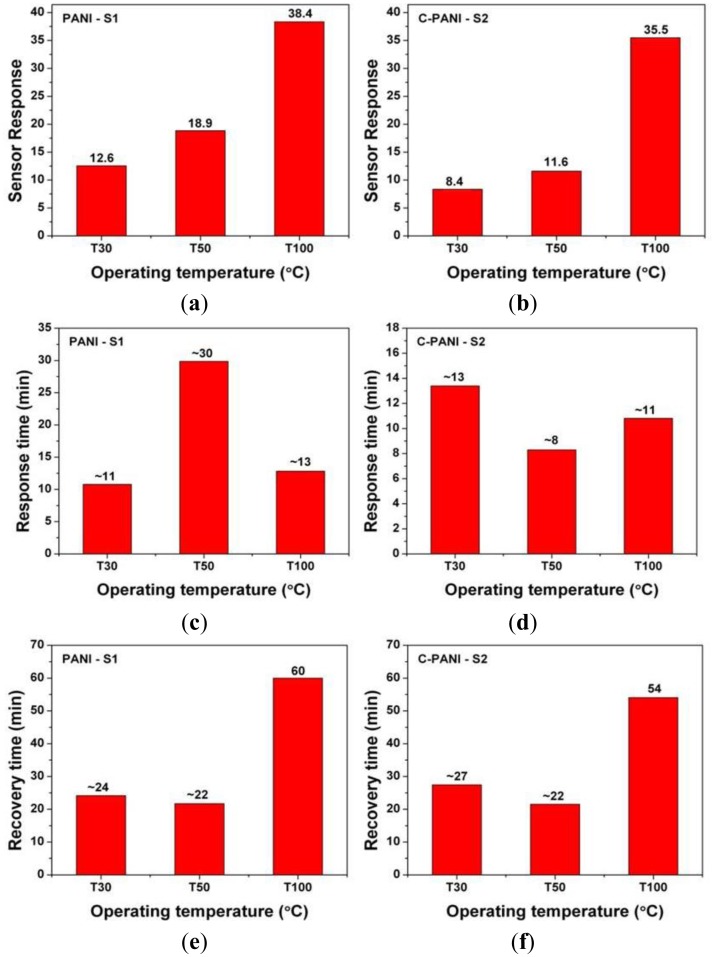
Toluene sensing behavior for PANI (S1) and C-PANI (S2) at different operating temperatures (**a**,**b**) Sensor response (**c**,**d**) Response time and (**e**,**f**) Recovery time.

Based on the sensing behavior data plotted in Figure 6, the sensor response for sample S1 and S2 can be calculated with the following formula [46,47]:

(1) $$\text{Sensing response}=\frac{\left(R_{a}-R_{g}\right)}{R_{a}}\times 100$$

where *R_a_* is a film resistance in air and *R_g_* is a film resistance in presence of the test gas. Figure 7 shows the extracted sensor parameters in terms of sensor response, response time and recovery time. The response time and recovery time is defined as 90% of the time taken for the total resistance change in the presence of test gas and to recover original base resistance after desorption of the residual gases, respectively [7,17,47,48].

As it can be observed in Figure 7a,b; the sensor response of homogeneous PANI (S1) and C-PANI (S2) sensing film increases with the operating temperature. However, the response time behavior as well as recovery time behavior of both the polymer films is quite different. In the case of response time in Figure 7c,d, the lack of any particular trend is visible. A similar case is also observed for the recovery time of both the samples S1 and S2 in Figure 7e,f. The response time for sample S1 (∼11 min) and S2 (∼8 min) are lowest at 30 and 50 °C, respectively. The recovery time for sample S1 (∼22 min) and S2 (∼22 min) is lowest at 50 °C. The reason for the high response time is due to the inability of the sensors to reach saturation in the resistance change and it can be attributed to large active surface area for the sensor (60 mm × 40 mm). However, after waiting for 10 min of the toluene admittance, the response time seems to be quite high. The high recovery time can be attributed to the room temperature operation and inability for desorption of chemisorbed analyte only by flushing N_2_ gas (at 1 SCCM).

## 4. Conclusions

To conclude, the present work discusses and compares the sensing behavior of polyaniline (PANI) and graphene/PANI nanocomposite (C-PANI) films as toluene (C_6_H_5_–CH_3_) gas sensors. The polymer films are grown by LbL spin coating PANI-NMP and graphene-PANI-NMP solutions. The spin-coated films are annealed at 230 °C and sputtered with aluminum (Al) to obtain electrodes. PANI and C-PANI films are characterized using SEM as well as FTIR and their sensing behavior towards 100 ppm of toluene analyzed at different operating temperatures of 30, 50 and 100 °C. The sensor response of both the polymer samples is found to increase with the temperature. However considering the response time and recovery time behavior; C-PANI films at the operating temperature of 50 °C shows better sensing behavior.

## Figures and Tables

**Table 1.** Toluene sensing using resistive gas sensor with different sensing materials.

**Sensing Materials**	**Additives/Catalysts**	**Resistance (R) in Presence of Analyte**	**Detection Range**	**Operating Temperatures**	Sensitivity*	**Ref.**
Nanoporous TiO_2_	Pd	decreases	50–200 ppm	RT	1.85 for 200 ppm	[12]
WO_3_ microtubes	Carbon	decreases	50–500 ppb	90 °C	39 for 500 ppb	[13]
ZnO and TiO_2_-doped ZnO nanostructures	TiO_2_	decreases	1–3000 ppm	160–390 °C	16.10 for 100 ppm (at 290 °C)	[7]
TiO_2_ nanostructured films by hydrothermal method	–	decreases	50 ppb	450–550 °C	24 for 50 ppm for 10 min exposure (at 500 °C)	[14]
WO_3_ using cotton fibers as templates	Carbon	decreases	100 ppb–1000 ppm	190–370 °C	0.8 for 100 ppb for 40 sec exposure (at 320 °C)	[15]
TiO_2_ nanotubular films by hydrothermal method	–	decreases	50 ppm	500 °C	51% for 50 ppm toluene (at 500 °C)	[6]
Pure and Sn-, Ga- and Mn-doped ZnO nanoparticles	Sn, Ga and Mn	decreases	5000 ppm	200–600 °C	1050 to 5000 ppm for Mn-doped ZnO (at 400 °C)	[16]
NiO crystallites by hydrothermal method	–	increases	3–1100 ppm	350 °C	1.28 for 11 ppm and 2.2 for 1100 ppm	[17]
Tetrapod-shaped ZnO nanopowders	–	decreases	100 ppm	180–480 °C	11 for 100 ppm (at 320 °C)	[18]
Carbon nanoparticles (CNP)/N,N,- dimethyl-1,3-propanediamine-copolymer	Carbon black	increases	<550 ppm	30 °C	0.04 for 200 ppm	[11]
Hybrid film of chemically modified graphene and vapor-phase-polymerized PEDOT	Graphene	increases	Fully saturated	RT	0.3 for fully saturated	[9]

* As definition of sensitivity varies in these studies, the sensitivity is normalized as (R_final_–R_base_)/R_base_.
